# Expanding the Clinico-Genetic Spectrum of Myofibrillar Myopathy: Experience From a Chinese Neuromuscular Center

**DOI:** 10.3389/fneur.2020.01014

**Published:** 2020-09-15

**Authors:** Yue-Bei Luo, Yuyao Peng, Yuling Lu, Qiuxiang Li, Huiqian Duan, Fangfang Bi, Huan Yang

**Affiliations:** ^1^Department of Neurology, Xiangya Hospital, Central South Hospital, Changsha, China; ^2^Department of Neurology, the First Affiliated Hospital of Guangxi Medical University, Nanning, China

**Keywords:** myofibrillar myopathy, desminopathy, titinopathy, BAG3opathy, filaminopathy, FHL1opathy

## Abstract

**Background:** Myofibrillar myopathy is a group of hereditary neuromuscular disorders characterized by dissolution of myofibrils and abnormal intracellular accumulation of Z disc-related proteins. We aimed to characterize the clinical, physiological, pathohistological, and genetic features of Chinese myofibrillar myopathy patients from a single neuromuscular center.

**Methods:** A total of 18 patients were enrolled. Demographic and clinical data were collected. Laboratory investigations, electromyography, and cardiac evaluation was performed. Routine and immunohistochemistry stainings against desmin, αB-crystallin, and BAG3 of muscle specimen were carried out. Finally, next-generation sequencing panel array for genes associated with hereditary neuromuscular disorders were performed.

**Results:** Twelve pathogenic variants in *DES, BAG3, FLNC, FHL1*, and *TTN* were identified, of which seven were novel mutations. The novel *DES* c.1256C>T substitution is a high frequency mutation. The combined recessively/dominantly transmitted c.19993G>T and c.107545delG mutations in *TTN* gene cause a limb girdle muscular dystrophy phenotype with the classical myofibrillar myopathy histological changes.

**Conclusions:** We report for the first time that hereditary myopathy with early respiratory failure patient can have peripheral nerve and severe spine involvement. The mutation in Ig-like domain 16 of *FLNC* is associated with the limb girdle type of filaminopathy, and the mutation in Ig-like domain 18 with distal myopathy type. These findings expand the phenotypic and genotypic correlation spectrum of myofibrillar myopathy.

## Introduction

Myofibrillar myopathy (MFM) is a group of hereditary neuromuscular disorders characterized by dissolution of myofibrils and abnormal intracellular accumulation of proteins, which are the constitutive or functional components of the Z disc. The defining morphological features of MFM are streaming, thickening, or dissolution of Z disc on electron microscopy. Other characteristic light microscopic changes are eosinophilic materials, rimmed vacuoles, and amorphous deposits and rubbed out fibers. Despite the common histological features, significant variation exists in each subtype in terms of clinical manifestation and molecular basis.

There is an ever-expanding panel of genes associated with myofibrillar myopathies including *DES, CRYAB, MYOT, LDB3, FLNC, BAG3, FHL1, TTN, PYROXD1* and *KY*, which encode proteins that are the integral part of or functionally associated with Z disc (1). Meanwhile, there are case reports in which other mutated genes cause histological changes compatible with myofibrillar myopathy. These genes include *ACTA1, HSPB8, PLEC, DNAJB6*, and *LMNA* (2–6). The majority of MFM patients follow an autosomal dominant inheritance pattern, while less frequently, the disease is transmitted by autosomal recessive or X-linked dominant/recessive pattern (7–9). There are several case reports of Chinese MFM patients, and one study identified a founder mutation in *FLNC* gene in 34 patients from Hong Kong area (10–13). In this study, we present the clinical, histological, immunohistochemical, and genetic analysis in 18 Chinese MFM patients diagnosed in our neuromuscular center. Seven novel mutations in *DES, FLNC, FHL1*, and *TTN* have been identified.

## Patients and Methods

### Patients

Between 2012 and 2019 in the Department of Neurology, Xiangya Hospital, 17 patients were diagnosed of MFMs based on myopathological findings including the following: eosinophilic bodies on eosin and hematoxylin (HE) staining, cytoplasmic bodies, rimmed vacuoles on modified trichrome Gomori staining and positive sarcoplasmic immunostaining for MFM-related proteins, and exclusion of other diseases that could demonstrate eosinophilic bodies or rimmed vacuoles, e.g., inclusion body myositis, on clinical features. One patient was excluded because of decline of gene test. Two additional patients were diagnosed as MFM based on genetic pedigree analysis (patient 2 was the younger brother of patient 1, patient 7 was patient 6's father) and clinical symptoms. As a result, 18 patients were recruited for this study. All recruited patients have signed consent forms.

### Clinical Data

Demographic and clinical data were collected. Laboratory investigations including blood routine, serum CK levels, electrocardiogram, echocardiography, and electromyography were performed.

### Histology and Histochemistry

Muscle specimens of biceps brachii or quadriceps femoris of 16 patients were snap frozen by isopropene cooled in liquid nitrogen. Sections of 8 μm thickness were cut using a cryostat (Leica CM1900). Routine stainings were performed as follows: HE, modified trichrome Gomori, NADH, SDH, COX/SDH double stain, acid phosphatase, oil red, PAS, and ATPase (pH 4.2, 4.6, and 9.6). Histological changes such as necrotic and regenerating fibers, eosinophilic bodies, and amorphous deposits were counted in six random fields under 200× magnification.

### Immunohistochemical Studies

Ten micrometer thick serial sections were cut for immunohistochemistry studies. Biopsies from subjects, who were ultimately deemed to be free of muscle diseases, were used as normal controls. Sections were blocked by 0.3% hydrogen peroxide in methanol and 10% goat serum in PBS for 30 min each, then incubated in primary antibodies against desmin (Abcam, 1:400), alpha-B crystalin (Abcam, 1:500), and bcl-2 associated athanogene 3 (BAG3, Abcam, 1:200) overnight at 4°C. After rinsing in PBS, sections were incubated in biotinylated secondary antibodies for 30 min. The Vectastain ABC kit (Vector Laboratories, CA) was used for immunodetection. After developed by DAB, the tissues were counterstained by hematoxylin for 10 s, then dehydrated through graded ethanol, cleared in xylene, and finally mounted by resin. The numbers of fibers with focal areas of increased reactivity for desmin, alpha-B crystalin, and BAG3 were counted in six random fields under 200× magnification.

### Genetic Studies

Genomic DNA was isolated from peripheral blood (MyGenostics, Beijing) of all 18 cases. Samples were pooled and sequenced on HiSeq X Ten (Illumina, CA) using 2 × 150 paired end sequencing. Sequences were aligned to the human genome reference (hg19) sequence using the Burrows-Wheeler Alignment tool (BWA 0.7.12) with default parameters. Detected sequence variants, if present in the dbSNP, HapMap, 1,000 Genome, ESP6500, ExAC, or in-house Chinese Exome Database (1,500 Chinese Han individuals), were all removed. Deleterious SNVs were predicted by SIFT (sift.bii.astar.edu.sg/), Polyphen-2 (genetics.bwh.harvard.edu/pph2/), and MutationTaster (www.mutationtaster.org/) programs. Candidate SNVs were validated by ABI3730 sequencer. In particular, genes associated with MFM, including *FHL1, FLNC, CRYAB, BAG3, DES, MYOT, LDB3, DNAJB6*, and *LMNA* were included in the panel. The two BAG3opathy patients and patient 14 were also screened for *PMP22* duplication or deletion by multiplex ligation-dependent probe amplification and genes associated with Charcot-Marie-Tooth disease (CMT) by targeted next-generation sequencing to exclude coexisting CMT.

## Results

### Overview

In the present study, 11 mutations were identified in *DES, BAG3, FLNC, FHL1*, and *TTN* (Table 1), of which 7 were not reported previously. The causative gene for patient 18 remained elusive despite extensive screening for the known genes for hereditary neuromuscular disorders.

**Table 1:** Genetics of the present MFM patient cohort.

**Patient no**.	**Gene**	**Chromosome**	**Exon**	**Transcript no**.	**Nucleotide**	**Protein**	**Reference**
1	*DES*	2	7	NM_1927	c.1256C>T	p.Pro419Leu	None
2	*DES*	2	7	NM_1927	c.1256C>T	p.Pro419Leu	None
3	*DES*	2	7	NM_1927	c.1256C>T	p.Pro419Leu	None
4	*DES*	2	7	NM_1927	c.1256C>T	p.Pro419Leu	None
5	*DES*	2	6	NM_1927	c.1096_1098delACA	p.Asn366del	(14)
6	*DES*	2	6	NM_1927	c.1096_1098delACA	p.Asn366del	(14)
7	*DES*	2	6	NM_1927	c.1096_1098delACA	p.Asn366del	(14)
8	*DES*	2	6	NM_1927	c.1076_1077ins GGCCAGTGG	p.Glu359delins GluAlaSerGly	None
9	*BAG3*	10	3	NM_004281	c.626C>T	p.Pro209Leu	(15)
10	*BAG3*	10	3	NM_004281	c.626C>T	p.Pro209Leu	(15)
11	*FLNC*	7	36	NM_001458	c.6004+3G>A	splicing	None
12	*FLNC*	7	33	NM_001458	c.5468C>T	P.Thr1823Met	None
13	*FHL1*	X	5	NM_001159702	c.386G>A	p.Cys129Tyr	None
14	*TTN*	2	344	NM_001267550	c.95134T>C	p.Cys31712Arg	(16–22)
15	*TTN*	2	344	NM_001267550	c.95185T>C	p.Trp31729Arg	(23)
16	*TTN*	2	69	NM_001267550	c. 19993G>T	p.Glu6665X	None
			363	NM_001267550	c. 107545delG	p.Ala35849Glnfs*16	None
17	*TTN*	2	69	NM_001267550	c. 19993G>T	p.Glu6665X	None
			363	NM_001267550	c. 107545delG	p.Ala35849Glnfs*16	None
18	None	-	-	-	-	-	-

The clinical features were summarized in Table 2. There was a male predominance with a male to female ratio of 1.6:1. The age of disease onset ranged from 1 to 48 years (mean ± SD = 25.0 ± 16.3 years) with duration from 1 to 27 years (10.6 ± 8.1 years). Except for one filaminopathy patient who presented with finger muscle atrophy, all cases demonstrated a more severe involvement of the lower limbs. Half of the patients demonstrated a pattern of mixed proximal and distal weakness and 27.8% had predominantly distal weakness. Of note, all four patients with *TTN* mutations displayed a selective tibialis anterior involvement. Seventy-seven-point-eight percent of patients showed muscle wasting, 33.3% experienced prolonged dyspnea. Dysphagia/dysphonia was present in 16.7% of patients. Joint abnormalities were found in 35.3% of patients, including joint contractures, scoliosis and rigid spine. Serum creatine kinase levels were mildly to moderately elevated (700.8 ± 440.3 U/L).

**Table 2:** Clinical features of the present MFM patient cohort.

**Patient no**.	**Gender**	**Age of onset (year)**	**Duration (year)**	**Weakness pattern**	**Joint contractures**	**CK (U/L)**	**Cardiac evaluation**
1	M	37	5	Lower proximal	–	747	PI/right heart + LA enlargement
2	M	33	8	Upper + lower proximal + distal	–	935.7	PI/LA enlargement
3	M	33	3	Lower distal	–	1366	Frequent APB + CRBBB + LAFB/LA enlargement
4	F	45	8	Upper + lower proximal + distal	–	383.4	NA
5	M	42	1	Lower distal	–	227.7	CRBBB
6	M	30	6	Lower proximal	–	1568.2	CRBBB/LA enlargement
7	M	48	19	Upper + lower proximal + distal	–	75.3	PI
8	M	13	20	Upper + lower proximal + distal	–	1016.5	Normal
9	F	5	20	Lower distal	Achilles tendon/rigid spine	374.2	Obstructive hypertrophic cardiomyopathy
10	F	9	10	Lower proximal + distal; scapular winging	Achilles tendon/talipes cavus/scoliosis	1269.7	Mild mitral + tricuspid + pulmonary valve regurgitation
11	M	37	10	Upper distal	MCP/PIP/elbow/scoliosis	691.3	NA
12	F	35	6	Lower proximal	–	259.2	Normal
13	F	6	2	Lower proximal + distal	–	450.8	Mild mitral + tricuspid regurgitation
14	M	42	10	Upper + lower distal	–	302.1	LAFB/LA enlargement
15	M	15	5	Upper + lower proximal + distal	Achilles tendon/scoliosis	340.5	Atrial septal defect-closed
16	F	1	27	Lower proximal + distal	Talipes cavus/scoliosis	375.1	Mild mitral + tricuspid + pulmonary valve regurgitation
17	F	1	26	Lower proximal + distal	Talipes cavus/scoliosis	296	Normal
18	M	18	5	Lower proximal	–	993.2	Mild mitral + tricuspid regurgitation

*CRBBB, complete right bundle branch block; LA, left atrium; LAFB, left anterior fascicular block; NA, not available; PI, pacemaker implant*.

Of the 16 patients who underwent heart assessment, 13 exhibited cardiac involvement. Both cardiac structural and electrophysical abnormalities were found in 31.3% of cases, 37.5% had only structural changes, and 12.5% only arrhythmia. The types of arrhythmias included bundle branch block and atrial/ventricular premature beat. Structural heart abnormalities included ventricle thickening, atrium enlargement, and valve regurgitation. The atrial septal defect in patient 17 was considered incidental.

NCS and EMG were performed in 17 patients. Nine patients (52.9%) demonstrated pure myogenic changes including small MUAPs, early recruitment, and polyphasia. Of these, one patient with *FLNC* mutation and one with *FHL1* mutation also showed myotonic discharges. Four patients (23.5%) showed mixed myopathic and neuropathic features. Seven patients (41.2%) demonstrated peripheral nerve involvement, of which six were consistent with an axonal type. Motor nerves were preferentially involved in these patients (Table 3). Findings on muscle pathology were summarized in Table 4 and presented in the following sections.

**Table 3:** Electromyographic features of the present MFM patient cohort.

**Patient**	**Spontaneous activity**	**MUP**			**Myotonic discharges**	**CMAP reduction**	**SNAP reduction**	**MCV slowing**	**SCV slowing**
		**Large**	**Small**	**Large + small**					
1	✓		✓	✓		✓		✓	
2	NA	NA	NA	NA	NA	NA	NA	NA	NA
3	✓		✓						
4	✓		✓			✓	✓	✓	
5	✓		✓			✓			
6	✓		✓						
7			✓						
8			✓						
9	✓			✓		✓	✓	✓	✓
10	✓	✓				✓	✓	✓	✓
11	✓			✓		✓			
12	✓		✓		✓				✓
13	✓		✓		✓	✓			
14	✓	✓				✓	✓	✓	
15			✓						
16			✓						
17	✓		✓			✓			
18		✓				✓		✓	

**Table 4:** Myopathological changes of the present MFM patient cohort.

**Patient no**.	**Necrosis (%)**	**Regeneration (%)**	**Central nuclei (%)**	**Eosinophilic bodies (%)**	**Cytoplasmic bodies (%)**	**Amorphous deposits (%)**	**Non-rimmed vacuoles (%)**	**Rimmed vacuoles (%)**	**Rubbed out fibers (%)**	**Desmin (%)**	**BAG3 (%)**	**αB crystallin (%)**	**Interstitial proliferation**
1	0.9	0.5	15.0	6.6	0.5	16.0	0.2	0.1	5.6	12.8	9.2	14.1	+
2	NA	NA	NA	NA	NA	NA	NA	NA	NA	NA	NA	NA	NA
3	2.9	2.1	22.2	3.1	2.5	3.8	11.5	3.5	6.5	19.7	3.8	3.3	++
4	1.1	0.9	9.5	1.5	0.4	4.9	2.5	1.5	6.8	5.5	1.8	1.5	–
5	0.1	0.4	38.4	6.0	0.6	5.1	0.4	0.0	5.3	9.0	3.6	3.4	+
6	0.2	0.3	11.1	2.2	1.1	4.1	0.2	0.0	2.3	3.7	3.1	4.6	+
*7*	NA	NA	NA	NA	NA	NA	NA	NA	NA	NA	NA	NA	NA
8	0.6	0.0	3.4	0.1	0.0	0.2	0.0	0.0	0.1	1.0	0.1	0.2	++
9	0.3	0.1	1.0	0.5	6.7	6.8	0.0	0.0	0.8	6.4	5.7	6.5	–
10	0.4	0.0	1.8	2.4	1.4	2.2	0.4	0.0	2.0	4.3	3.6	2.3	++
11	0.6	0.4	12.4	0.0	0.0	0.0	0.4	1.8	0.0	0.0	0.0	0.0	++
12	0.3	0.2	18.5	0.0	0.0	0.0	0.9	0.6	0.0	0.0	0.0	0.0	–
13	0.1	0.0	10.0	2.4	4.4	1.0	0.0	0.0	0.0	0.2	0.0	0.5	+
14	1.3	0.9	31.4	1.1	4.5	0.0	0.2	0.0	1.4	5.0	1.8	3.9	+
15	0.0	0.2	10.5	0.2	0.0	0.0	0.0	0.0	0.4	0.8	0.3	0.0	+
16	0.5	1.1	86.8	0.1	0.3	0.3	2.6	4.0	0.9	4.3	1.8	0.0	+
17	1.3	0.6	79.3	0.4	0.6	11.9	2.9	0.3	0.0	8.8	1.5	0.8	+
18	2.7	0.7	36.7	1.8	0.0	2.0	2.0	0.0	0.7	1.5	1.1	1.3	++

*−, no interstitial proliferation; +, ++ corresponds to mild, moderate interstitial proliferation*.

### Desminopathy

**Figure 1:**
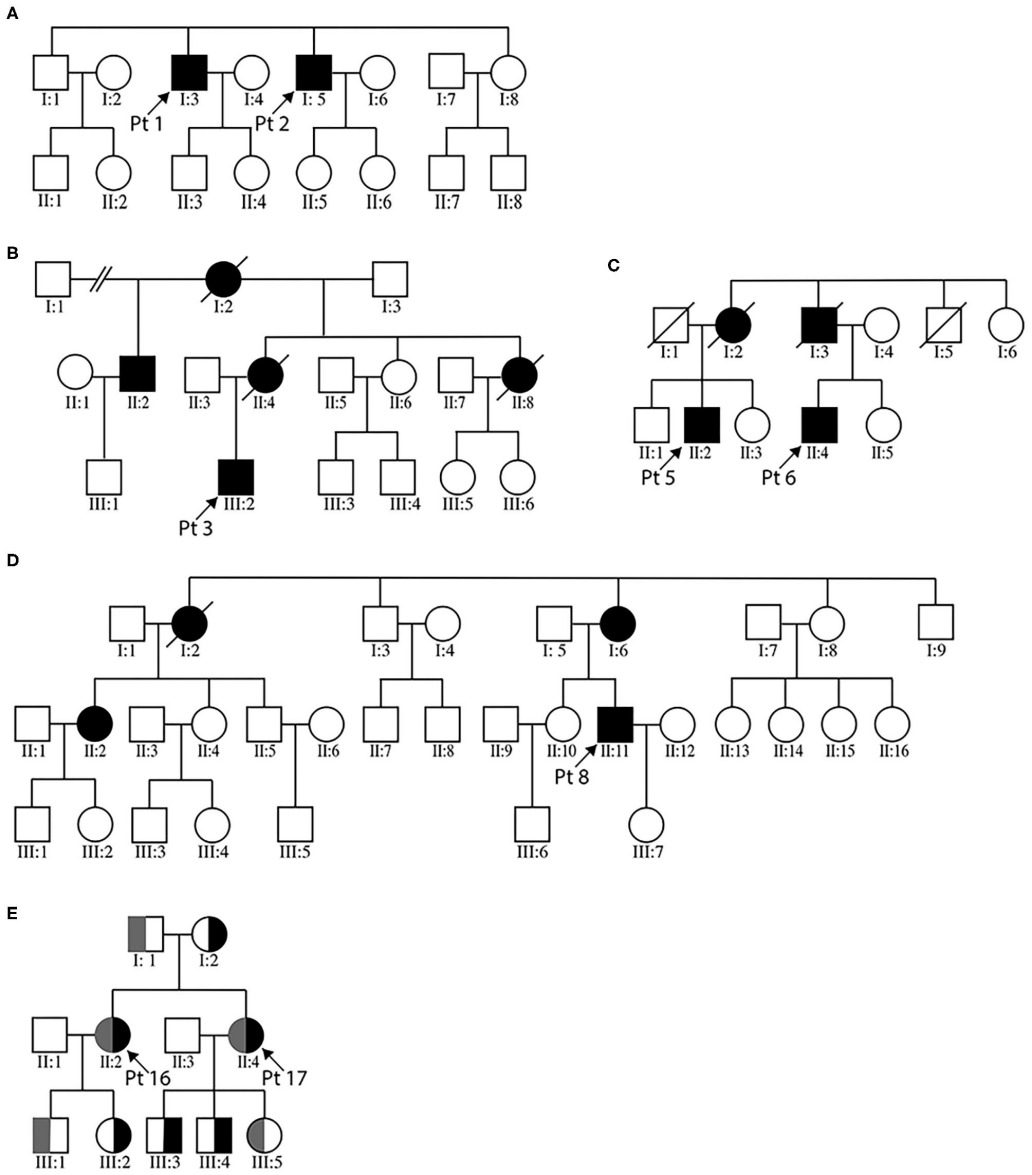
Pedigree charts of the enrolled MFM patients. **(A–D)** illustrate the three desminopathy families. **(E)** demonstrates the family tree of patients 16 and 17. For panel **(E)**, gray bar represents the c. 107545delG mutation carrier, black bar the c. 19993G>T carrier.

There were four pedigrees with *DES* mutations (Figures 1A–D), and the inheritance pattern was consistent with an autosomal dominant mode. The disease tended to present in adulthood (age of onset 35.1 ± 10.9 years). All eight patients demonstrated lower extremity weakness, four also had upper limb weakness. Three patients had heart pacemaker implantation. Patient 3 also had episodic palpitation and syncope. His grandmother on mother's side, mother, and aunt all had sudden death of presumable “heart problems.” His half-uncle on mother's side had similar lower limb weakness in his thirtieth (II:2 of Figure 1B). Regardless of disease duration, none exhibited joint contractures. The seven patients who finished EMG studies all showed myogenic changes, and the one with mixed myogenic and neurogenic changes was shown to have axonal polyneuropathy with motor nerve involvement. Three cases showed significant structural changes of heart (Table 2). Apart from the three patients with heart implantation, another three showed arrhythmia including atrial premature beat, right bundle block, and fascicular block. On muscle biopsy, the fibers with eosinophilic bodies ranged from 0.1 to 6.6%, rimmed vacuoles from null to 3.5%, rubbed out fibers from 0.1 to 6.8%.

Next-generation sequencing of patients 1 and 2 (brothers, Figure 1A) revealed two candidate mutations, c.772C>T in *BAG3* and c.1256C>T in *DES*. The *BAG3* variant was previously reported in an individual with long QT interval but no muscle symptoms (24). The *DES* c.1256C>T variant was also identified in patients 3 and his affected half-uncle (Figure 1B), as well as in patient 4. This variant was absent in the unaffected siblings and children of patient 1 and 2, as well as in the parents of patient 4. It causes replacement of a conserved proline by leucine. This substitution is listed as of uncertain significance by ClinVar database and is predicted to be probably damaging by PolyPhen-2. Based on the homogenous phenotype of these patients, we propose that the *DES* c.1256C>T substitution is more likely the causative mutation. It is worth mentioning that the *BAG3* c.772C>T variant was also found as the only possible pathogenic variant in another patient from our department, who has proximal limb weakness, scoliosis, and scapular winging. Muscle morphology was of mild myopathic changes and lack of any characteristic MFM changes (data not shown). We could not definitively negate the pathogenicity of this variant.

### BAG3opathy

**Figure 2:**
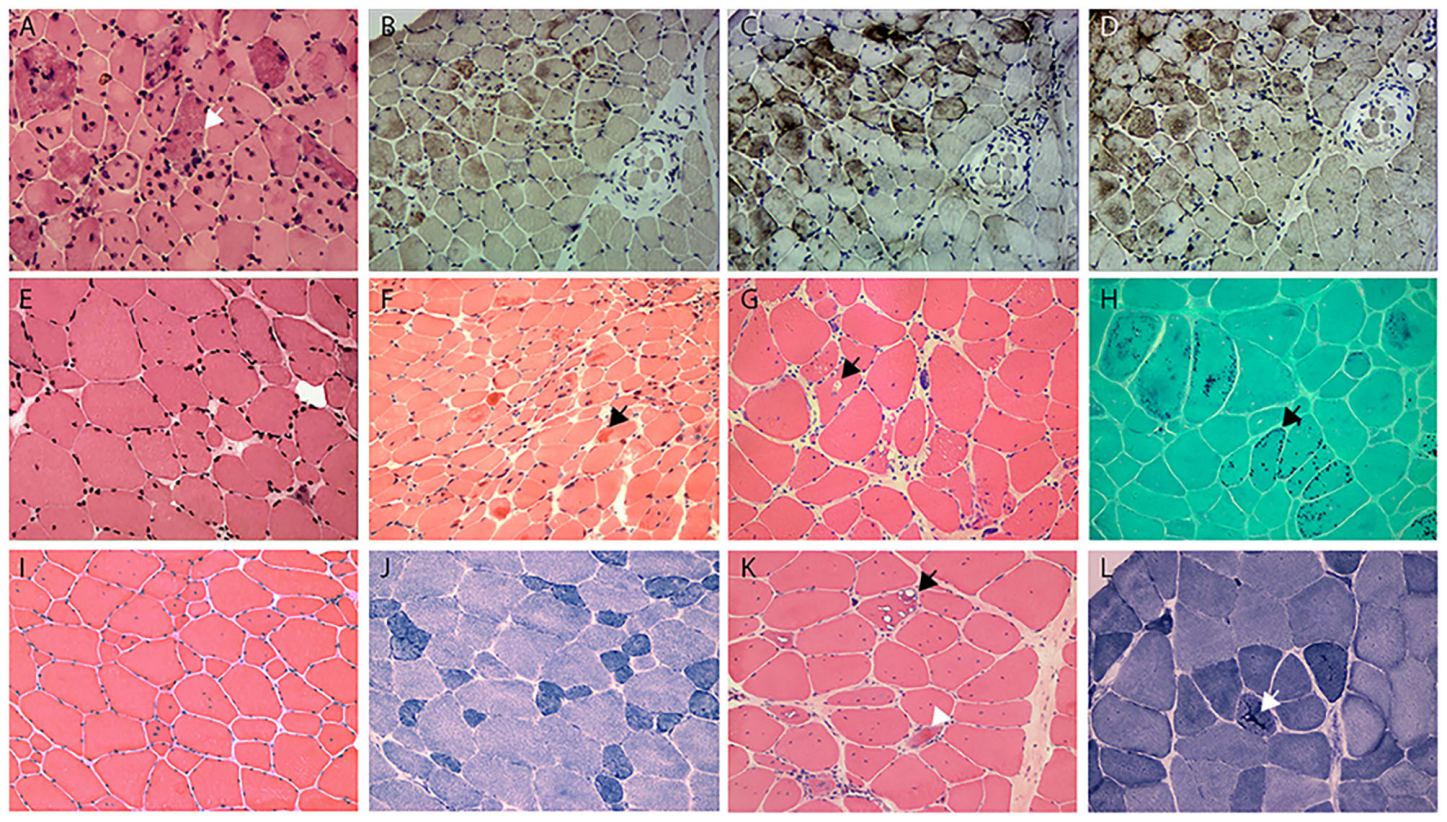
**(A)** HE staining shows the numerous intramuscular eosinophilic deposits in BAG3opathy (arrow, patient 9); **(B–D)** immunohistochemistry of serial sections from patient 9 shows aggregation of MFM-related proteins [**(B)**, desmin; **(C)**, BAG3; **(D)**, αB crystallin; different field from **(A)**], eosinophilic aggregates were most strongly immunoreactive with BAG3 in this patient. Note the aggregates concentrated at the upper myofascicle, while the lower right fascicle appeared normal. **(E)** HE staining shows fiber size variation and rare regeneration in filaminopathy (patient 11). **(F)** HE demonstrates eosinophilic materials in FHL1opathy (arrow, patient 13). Note the overall small fiber size. **(G)** HE shows vacuolated fibers (arrow), central nucleated fibers, and nuclear clumps in HMERF (patient 15). **(H)** Gomori shows fibers with necklace cytoplasmic bodies (arrow, patient 14). **(I,J)** HE and NADH demonstrates increased central nuclei and selective type 1 atrophy in titinopathy (patient 16). **(K)** HE shows rimmed vacuoles (arrow) and esosinophilic bodies (arrowhead) in titinopathy (patient 17). **(L)** NADH shows relative preservation of the myofibrillar network in the majority of fibers except for occasional bar-like enzyme aggregation (arrow) in patient 17. Magnification: ×200.

The two BAG3opathy patients carried the same c.626C>T mutation, as in accordance with most other BAG3opathy cases. They both presented in childhood and had severe lower limb weakness, especially distal muscles (MRC 2–3/5). Ten years into disease progression, patient 9 developed type 2 respiratory failure (pH 7.31, pO_2_ 78 mmHg, pCO_2_ 74 mmHg) induced by community-acquired pneumonia. Since then, she had been intermittently using noninvasive ventilator (Bi-level Positive Airway Pressure mode) at night (5–7 h per night for 3–7 days per week), as suggested by local pulmonologist. During hospitalization, she was also found to have obstructive hypertrophic cardiomyopathy. Both patients had axonal sensorimotor polyneuropathy confirmed by NCS and EMG (Table 3). Mildly to moderately increased positive sharp waves and large motor unit potentials (MUPs) were present in the upper and lower limb muscles of patient 10. Compound motor action potentials (CMAPs) of her tibial and common femoral nerves were not elicitable, neither were sensory nerve action potentials (SNAPs) of sural, median, and ulnar nerves. In patient 10, there were moderately to severely increased positive sharp waves and fibrillation potentials. She demonstrated large as well as small MUPs with minimal voluntary contractions. The recruitment was reduced. All tested sensory nerves were inexcitable, while CMAPs of motor nerves were of small amplitude. The nerve involvement was so extensive and severe that both patients were initially diagnosed as axonal CMT. Mild joint abnormalities, including contracted Achilles tendons and scoliosis, were noticed in both patients. Pathological changes of this group were similar to those with desminopathy, including eosinophilic bodies (0.5–2.4%), cytoplasmic bodies (1.6–6.7%), amorphous deposits (2.2–6.8%), and rubbed out fibers (0.8–2.0%; Figure 2A). Another feature of BAG3opathy patients was that these changes were conspicuous in focal areas while in other field the muscle may appear to be completely normal (Figures 2B–D).

### Filaminopathy

The disease presented at mid-thirtieth in both patients. Patient 11 first noticed atrophy of both hands with minimal difficulties in fine motor skills, and developed lower extremity weakness within 10 years. There was atrophy of his first dorsal interosseous muscles and tibialis anterior. He had mild contracture of metacarpophalangeal, proximal interphalangeal joints, and elbows, as well as mild scoliosis. Patient 12 complained of progressive leg weakness. Both patients exhibited mixed myogenic and neurogenic changes on EMG, but NCS was unremarkable. The myopathological changes were minimal (Figure 2E). Immunohistochemical staining against the three Z band associated proteins was unremarkable. Additional immunohistochemistry study using antibody against filamin C demonstrated filamin C aggregations in myofibers in the two patients, but not in FHLopathy or desminopathy patients (data not shown).

**Figure 3:**
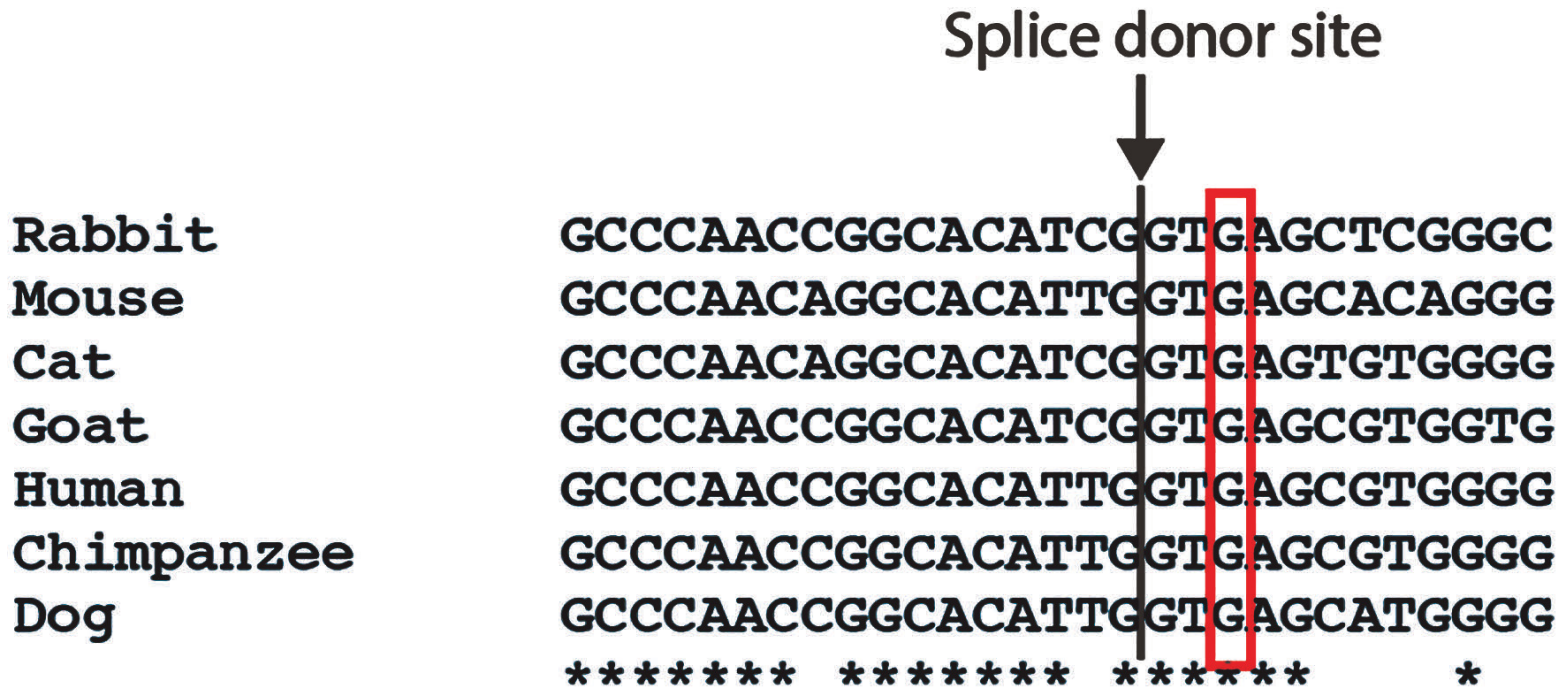
Comparison of the *FLNC* intronic c.6004+3 nucleotide among different species. *indicates the conserved nucleotide.

The intronic substitution c.6004+3G>A in patient 11 was not found in general population according to the Human Gene Mutation Database, and was conserved among species (Figure 3). It was right next to the donor splice site and likely to cause skipping of exon 36. The p.Thr1823Met missense mutation in patient 12 was present in 0.06% of general population according to gnomAD, yet was listed as variant of uncertain significance by ClinVar. It was predicted to be probably damaging by PolyPhen-2 (score 1.0), but was tolerated by SIFT. Blood samples of the parents were unfortunately unavailable as both were deceased.

### Titinopathy

Four tininopathy patients were included in the present study. The phenotype of patient 14 and 15 were in line with hereditary myopathy with early respiratory failure (HMERF). Patient 14 presented with distal lower extremity weakness in his early fortieth. Four years after disease onset, he developed nocturnal dyspnea and soon required noninvasive ventilation. There was marked reduction of CMAP and SNAP amplitudes with conduction velocity being normal. EMG demonstrated increased fibrillation potentials and large MUPs. The presenting symptom of patient 15 was progressive scoliosis and mild walking difficulty at age 15. Subsequent spinal fusion surgery at age 16 did not ameliorate his leg weakness. He developed post-exercise dyspnea at age 19. Pulmonary function test showed severe restrictive ventilatory defect and artery blood gas analysis revealed type II respiratory failure. Noninvasive ventilation was recommended. On muscle biopsy, the characteristic fibers with necklace cytoplasmic bodies (Figures 2G,H) were found in both patients. Two missense mutations in exon 344 of *TTN* (c. 95134T>C in patient 14, c.95185T>C in patient 15) were identified.

Patients 16 and 17 were sisters presenting with similar lower limb weakness. They learnt to walk at one and half years of age, and always ran more slowly than their peers. Despite the continuous progression of weakness, the two patients were still ambulatory at the time of biopsy. On physical examination, pronounced atrophy of quadriceps femoris, hamstrings, and tibialis anterior was noted. Both had lordosis and talipes cavus. Their mother had similar yet much milder lower limb weakness presenting in her 20th. She was still capable of sedentary work and ambulatory in her fiftieth. The father did not complain of any muscle symptoms and showed no obvious muscle atrophy. Of the third generation of this family, the second son of patient 17 (III:4), who was five years old, had frequent falls. Other children were asymptomatic. Muscle biopsies of biceps brachii from the two cases revealed pathological changes of different degrees. The main changes of patient 16 were increased central nuclei (10.5%) and selective type 1 atrophy (Figures 2I,J). In comparison, patient 17 demonstrated more severe changes including considerably more central nuclei (86.6%), eosinophilic materials (0.1%) and rimmed vacuoles (0.4%, Figure 2K). On NADH staining, the patients demonstrated occasional darkly stained bar-like area extending from vacuoles (Figure 2L). Overall, the titinopathy group had the highest numbers of central nuclei (52.0 ± 37.0%). The sisters harbored compound heterozygous mutations in *TTN* (Figure 1E). The allele with p.Glu6665X nonsense mutation originated from their mother, whereas the other allele with p.35849A>Qfs^*^16 mutation came from the father.

### Miscellaneous

Patient 13 managed to reach her developmental milestones until early childhood. Her parents noticed her having frequent falls and demonstrating a waddling gait from age 6 years. Physical examination revealed marked weakness of neck and lower limbs with preferential involvement of tibialis anterior. Biopsy of biceps (Figure 2F) showed central nuclear fibers (10%), eosinophilic bodies (2.4%), and cytoplasmic bodies (4.4%). Myofibers with desmin aggregates accounted for only 0.2% of total fibers. An unreported variant (c.386G>A) in *FHL1* gene was identified. This missense mutation caused substitution of a cysteine for a tyrosine at amino acid position 129, which was predicted to be probably damaging (score 0.999) according to PolyPhen-2.

Patient 18 was the only case with unidentified mutation in this study. He presented with lower limb weakness in young adulthood. He subsequently developed mild dysphagia and quadriceps atrophy. Nerve conduction studies revealed motor axonal neuropathy. His echocardiography at age 23 showed mild mitral and tricuspid regurgitation. Increased central nucleated fibers (36.7%), occasional eosinophilic bodies (1.8%), and fibers focally immunoreactive to desmin (1.5%), αB crystallin (1.1%), and BAG3 (1.3%) were found on muscle biopsy.

## Discussion

Since the main pathological event in MFM is considered disintegration of the Z disc, we first present a brief summary on its physiological features with emphasis on MFM-related proteins. The Z disc, whose core structure is formed by α-actinin homodimers, defines the boundaries of a sarcomere unit and provides an anchoring point for sarcomeres by cross-linking the neighboring actin thin filaments. The interaction between α-actinin and actin itself does not suffice to maintain proper contractile functions of sarcomeres. Other integral Z disc proteins also play a part in the Z disc-thin filament connection. For example, ZASP and myotilin are associated with α-actinin and actin, respectively (25, 26). The large protein titin binds to α-actinin and myosin on each terminal, thus serves as an elastic anchor for thick filaments to the Z disc. Another binding partner for titin is FHL1, which is associated with the I band part of titin and acts as a part of the mechanosensing machinery (27). Titin also interacts with filamin C to participate in stabilization of the Z disc (28). Filamin C in turn associates with actin, which strengthens the Z disc-thin filament connection. Desmin is one of the most important intermediate filament proteins in striated muscles. It links the Z disc and the costamere complex so as to stabilize the Z disc, and also links the nucleus to cytoskeleton network (29). Not only the innate defects of the Z disc-associated proteins lead to MFM pathology, disturbance of the turnover homoeostasis of these constitutive proteins also shows similar pathogenicity. Under both physiological and stress conditions, the small heat shock protein αB crystallin binds to titin to retain appropriate conformation of the latter and prevent it from denaturation (30, 31). The chaperone activity of αB crystallin also enables it to assist desmin scaffold assembly (32). BAG3 is a co-chaperone molecule involved in the protein quality control system, and is dedicated to clearance of aberrant protein aggregates by means of chaperone-assisted selective autophagy (CASA) and macrophagy (33–35). It has been shown that BAG3 interacts with αB crystallin and prevents mutant αB crystallin aggregation (36). Another co-chaperone DNAJB6 interacts with CASA complex that includes BAG3, the exact physiological significance of which requires further exploration (5).

In this retrospective study, we have described the clinical, electrophysiological, pathological, and genetic characteristics of 18 MFM patients at our neuromuscular clinic. Symptoms of desminopathy and filaminopathy tend to present in adulthood. In comparison, BAG3opathy cases have childhood onset, while titinopathy patients demonstrate a wider range of onset age from infancy to adulthood. Joint involvement is more prominent in BAG3opathy and titinopathy cases, yet is not a feature of desminopathy. Our desminopathy patients exhibit the most severe cardiac electrophysiological abnormalities, to the extent that three out of eight patients have undergone pacemaker implantation, whilst no cases of other genotypes require such procedure. Regardless of the genotypes, motor, or sensorimotor axonopathy is the predominant form of neuropathy in this cohort. BAG3opathy patients demonstrate the most severe peripheral nerve involvement. There are two reports of patients with the canonical MFM-related BAG3 mutation displaying a CMT plus rigid spine phenotype (37, 38). Neither patient manifests signs of cardiomyopathy, which is common among BAG3opathy. Moreover, despite the telltale finding of Z disc disarray in ultrastructural evaluation, no protein aggregation on light microscopy was reported in the two previous cases. In comparison, one of our BAG3opathy patients developed hypertrophic cardiomyopathy ten years after disease onset. Both of our BAG3opathy patients show the characteristic eosinophilic and cytoplasmic bodies, as well as Z disc protein aggregates. In the scenario of early onset, slowly progressive symmetrical distal weakness with paresthesia, and diffuse axonal changes on EMG/NCS, diagnosis of CMT should be made with caution as BAG3opathy can present with very similar manifestations. Proof of cardiac muscle involvement serves as a warning sign, and if present, muscle biopsy should be considered to seek for protein aggregation typical of BAG3opathy.

In terms of the molecular genetics of our cohort, *DES* is the most common gene linked to MFM, accounting for 44.4% of all cases, followed by *TTN* (22.2%). The types of mutation in *DES* include missense, deletion, and deletion/insertion. The novel c.1256C>T missense substitution seems to be a high frequency mutation with three families and one sporadic case in this study.

The inheritance pattern of *TTN* mutations can be autosomal dominant or recessive, or even the combination of both (9, 16, 39). In a large cohort of congenital titinopathy caused by autosomal recessive *TTN* mutations, axial weakness, early joint contractures, and progressive respiratory deficiency are the predominant clinical manifestations (40). The muscle pathology consists of increased central nuclei and cores/minicores, which is more indicative of congenital myopathy than MFM (40, 41). In another titinopathy cohort with autosomal recessive inheritance pattern, the patients present with either childhood onset generalized weakness or adult onset distal lower limb weakness (7). The coexistence of one dominantly and one recessively inherited mutations has been reported in several titinopathy cases with infantile to adult onset (9). The weakness pattern of these semi-dominant/recessive titinopathy cases is proximal and/or distal limb weakness, whilst pathology is myopathic with or without rimmed vacuoles. The dominant mutations are all tibial muscular dystrophy-related and are located in exon 363 (M-band exon 5), while the recessive mutations are all frameshifting. In the case of patients 16 and 17, the two sisters exhibit an infantile onset of limb girdle weakness, scoliosis plus the characteristic tibialis weakness without significant respiratory or cardiac insufficiency. Whilst the elder sister demonstrates a full picture of MFM pathology, the younger one only shows changes consistent with centronuclear myopathy, resembling the autosomal recessive titinopathy cases (40). Considering the remarkably similar phenotype between the two sisters, a different genetic background other than the compound heterozygous *TTN* mutations is unlikely. Again, we propose that different degrees of MFM pathology may coexist in the same patient and sampling bias may be the cause of discrepant morphological findings. According to the 2018 ENMC nomenclature consensus of limb girdle muscular dystrophy (LGMD) (42), patients16 and 17 could be diagnosed as “LGMD R10 titin-related.” Yet their inheritance pattern does not completely comply with an autosomal recessive manner. Instead, it follows a combined autosomal recessive and dominant pattern. The frameshifting c.107545delG mutation is situated in exon 363 and passed down by an autosomal recessive fashion, as carriers of this single variant in the family are all asymptomatic. That this variant is of benign nature is unlikely as it causes truncation of titin protein by 128 amino acids, which includes the 152nd immunoglobulin domain of titin. The dominantly inherited c.19993G>T nonsense mutation results in creation of a premature stop codon in the tandem immunoglobulin domain of the I-band part of titin. Whether this mutation is partially or completely penetrated needs further follow up of the third generation of this pedigree, as only one out of three offspring (III:4 of Figure 1E) carrying the nonsense mutation is manifesting.

So far, mutations associated with the HMERF phenotype are all located in exon 344 of *TTN*, which is in the 119th fibronectin type 3 region of the A-band part of titin. The c. 95134T>C missense mutation of patient 14 was first associated with HMERF in three Scandinavian families (16) and later in various ethnic groups including the Chinese population (17–23). It is noteworthy that in over 100 reported cases of HMERF, peripheral nerves have been considered spared in this phenotype (43). Our patient is the first HMERF patient that shows peripheral nerve involvement, which is consistent with an axonal sensorimotor polyneuropathy type. The recurrent c.95185T>C mutation patient 15 carries was first reported in one German HMERF family presenting with both proximal and distal weakness (23). Contracture of Achilles tendon and rigid spine has been reported in some HMERF cases (21), severe joint abnormalities are nevertheless not the predominant feature of HMERF. The early and severe involvement of spine in patient 15 is reminiscent of an Emery–Dreifuss muscular dystrophy phenotype, which has been reported in recessive titinopathy cases (44). Taken together, the titinopathy patients in the present study illustrate that: 1. the inheritance pattern of *TTN* is dependent on the malignant level of the mutation, which is at least partially determined by factors such as location, type, as well as the underlying pathomechanisms connected to the mutation; 2. titinopathy is a spectrum of disease entity with a plethora of combination of involved systems, disease manifestation and temporal progression, and pathologies.

At least three phenotypes have been associated with filaminopathy. Patients with the first form has an adult onset distal upper limb weakness with non-specific myopathic changes on muscle biopsy and lack of conspicuous intramuscular protein aggregation (45–47). Mutations in the N-terminal and Ig-like domain 15 of *FLNC* are related to this collective group. The second form is characterized by adult onset limb girdle weakness and the typical MFM pathology. So far mutations in Ig-like domains 7, 22, and 24 have been linked to this phenotype (48–50). Recently, a third filaminopathy phenotype, which is clinically delineated by restrictive cardiomyopathy and congenital myopathy, was reported (51). The causative mutations are in Ig-like domain 10. We report that the intronic variant possibly disrupting proper splicing of the region coding for Ig-like domain 18 is associated with the distal myopathy phenotype, and the novel c.5468C>T missense mutation in Ig-like domain 16 can cause the limb girdle phenotype.

## Conclusions

To conclude, in the present Chinese MFM cohort, desminopathy is the most common MFM subtype. The novel *DES* c.1256C>T substitution is a high frequency mutation. Sensorimotor axonopathy is the most common form of peripheral neuropathy in MFM patients. We confirm that BAG3opathy has the most severe peripheral nerve involvement, which can mimic CMT both clinically and electromyographically. We also find that combined recessive/dominant *TTN* mutations can cause a limb girdle muscular dystrophy phenotype with the characteristic MFM pathology. Patients with HMERF can have peripheral nerve, as well as severe spine involvement. The mutation in Ig-like domain 16 is associated with the limb girdle type of filaminopathy, and the mutation in Ig-like domain 18 with distal myopathy type. The pathogenicity of novel variants reported in this study requires further functional validation.

## Data Availability Statement

The datasets generated for this study can be found in the ClinVar database.

## Ethics Statement

The studies involving human participants were reviewed and approved by the Ethics Committee of Xiangya Hospital, Central South University. Written informed consent to participate in this study was provided by the participants and participants' legal guardian. Written informed consent was obtained from the individuals, and minor's legal guardian, for the publication of any potentially identifiable images or data included in this article.

## Author Contributions

Y-BL and HY designed this study. Y-BL wrote and HY revised this manuscript. YL and YP collected the data. FB, QL, and HD contributed to the muscle pathological evaluation. All authors contributed to the article and approved the submitted version.

## Conflict of Interest

The authors declare that the research was conducted in the absence of any commercial or financial relationships that could be construed as a potential conflict of interest.
