# Corrigendum: Expanding the Clinico-Genetic Spectrum of Myofibrillar Myopathy: Experience From a Chinese Neuromuscular Center

**DOI:** 10.3389/fneur.2020.636981

**Published:** 2021-01-08

**Authors:** Yue-Bei Luo, Yuyao Peng, Yuling Lu, Qiuxiang Li, Huiqian Duan, Fangfang Bi, Huan Yang

**Affiliations:** ^1^Department of Neurology, Xiangya Hospital, Central South Hospital, Changsha, China; ^2^Department of Neurology, the First Affiliated Hospital of Guangxi Medical University, Nanning, China

**Keywords:** myofibrillar myopathy, desminopathy, titinopathy, BAG3opathy, filaminopathy, FHL1opathy

In the original article, there was a mistake in Table 1 as published. For patients 16 and 17, the protein alteration resulting from the c. 107545delG mutation should be p.Ala35849Glnfs*16. The corrected Table 1 appears below.

**Table 1:** Genetics of the present MFM patient cohort.

**Patient no**.	**Gene**	**Chromosome**	**Exon**	**Transcript no**.	**Nucleotide**	**Protein**	**Reference**
1	*DES*	2	7	NM_1927	c.1256C>T	p.Pro419Leu	None
2	*DES*	2	7	NM_1927	c.1256C>T	p.Pro419Leu	None
3	*DES*	2	7	NM_1927	c.1256C>T	p.Pro419Leu	None
4	*DES*	2	7	NM_1927	c.1256C>T	p.Pro419Leu	None
5	*DES*	2	6	NM_1927	c.1096_1098delACA	p.Asn366del	(14)
6	*DES*	2	6	NM_1927	c.1096_1098delACA	p.Asn366del	(14)
7	*DES*	2	6	NM_1927	c.1096_1098delACA	p.Asn366del	(14)
8	*DES*	2	6	NM_1927	c.1076_1077ins GGCCAGTGG	p.Glu359delins GluAlaSerGly	None
9	*BAG3*	10	3	NM_004281	c.626C>T	p.Pro209Leu	(15)
10	*BAG3*	10	3	NM_004281	c.626C>T	p.Pro209Leu	(15)
11	*FLNC*	7	36	NM_001458	c.6004+3G>A	splicing	None
12	*FLNC*	7	33	NM_001458	c.5468C>T	P.Thr1823Met	None
13	*FHL1*	X	5	NM_001159702	c.386G>A	p.Cys129Tyr	None
14	*TTN*	2	344	NM_001267550	c.95134T>C	p.Cys31712Arg	(16–22)
15	*TTN*	2	344	NM_001267550	c.95185T>C	p.Trp31729Arg	(23)
16	*TTN*	2	69	NM_001267550	c. 19993G>T	p.Glu6665X	None
			363	NM_001267550	c. 107545delG	p.Ala35849Glnfs*16	None
17	*TTN*	2	69	NM_001267550	c. 19993G>T	p.Glu6665X	None
			363	NM_001267550	c. 107545delG	p.Ala35849Glnfs*16	None
18	None	-	-	-	-	-	-

The authors apologize for this error and state that this does not change the scientific conclusions of the article in any way. The original article has been updated.

